# Enhancement of the Catalytic Performance and Operational Stability of Sol-Gel-Entrapped Cellulase by Tailoring the Matrix Structure and Properties

**DOI:** 10.3390/gels8100626

**Published:** 2022-10-01

**Authors:** Corina Vasilescu, Simona Marc, Iosif Hulka, Cristina Paul

**Affiliations:** 1 Biocatalysis Group, Department of Applied Chemistry and Engineering of Organic and Natural Compounds, Faculty of Industrial Chemistry and Environmental Engineering, Politehnica University Timisoara, Carol Telbisz 6, 300001 Timisoara, Romania; 2 Laboratory of Magnetic Fluids, Center for Fundamental and Advanced Technical Research, Romanian Academy Timisoara Branch, Mihai Viteazu 24, 300223 Timisoara, Romania; 3 Faculty of Veterinary Medicine, University of Life Sciences “King Mihai I” from Timisoara, Calea Aradului 119, 300645 Timisoara, Romania; 4 Research Institute for Renewable Energy, Politehnica University Timisoara, Gavril Musicescu 138, 300501 Timisoara, Romania

**Keywords:** sol–gel entrapment, cellulase, magnetic nanobiocatalysts, catalytic performance, cellulose hydrolysis, reusability

## Abstract

Commercial cellulase Cellic CTec2 was immobilized by the entrapment technique in sol–gel matrices, and sol–gel entrapment with deposition onto magnetic nanoparticles, using binary or ternary systems of silane precursors with alkyl- or aryl-trimethoxysilanes, at different molar ratios. Appropriate tailoring of the sol–gel matrix allowed for the enhancement of the catalytic efficiency of the cellulase biocatalyst, which was then evaluated in the hydrolysis reaction of Avicel microcrystalline cellulose. A correlation between the catalytic activity with the properties of the sol–gel matrix of the nanobiocatalysts was observed using several characterization methods: scanning electron microscopy (SEM), fluorescence microscopy (FM), Fourier transform infrared spectroscopy (FT-IR) and thermogravimetric analysis (TGA/DTA). The homogeneous distribution of the enzymes in the sol–gel matrix and the mass loss profile as a function of temperature were highlighted. The influence of temperature and pH of the reaction medium on the catalytic performance of the nanobiocatalysts as well as the operational stability under optimized reaction conditions were also investigated; the immobilized biocatalysts proved their superiority in comparison to the native cellulase. The magnetic cellulase biocatalyst with the highest efficiency was reused in seven successive batch hydrolysis cycles of microcrystalline cellulose with remanent activity values that were over 40%, thus we obtained promising results for scaling-up the process.

## 1. Introduction

Environmental pollution that is caused by fossil fuels, the growing population, and the high costs of traditional energy sources are forcing researchers to develop new approaches to producing green and biodegradable energy resources [1]. In recent years, increased attention has been paid, among other issues, to reducing environmental pollution by the rapid elimination of volatile organic compounds using photothermal catalysis [2], as well as new strategies for the preparation of high-performance catalysts for the removal of pollutants [3] and the reduction of CO_2_ emissions [4].

Biomass, especially cellulose, is the most abundant biopolymer, and it is a low-cost energy source that can be degraded to produce biomaterials useful in many domains. Cellulose-hydrolyzing enzymes, such as cellulases, are catalysts that convert cellulose to glucose and are widely used in various applications in foods, cellulose and paper, detergents, textiles, agriculture, pharmaceuticals and medicine, but they are mainly for the production of biofuel [1]. Cellulose-degrading enzymes generally refer to a set of enzymes [5], the so-called *cellulase enzyme complex*, that are composed of endoglucanases (EC 3.2.1.4), exoglucanases (EC 3.2.1.91) and β-glucosidases (EC 3.2.1.21), that act synergistically to bioconvert cellulose to glucose, which is a precursor for the production of various added-value products [6,7].

The use of enzymes is challenging due to their low stability and activity along with their high cost and fragile nature. To overcome these disadvantages, a possible solution is to use immobilization, which also allows the biocatalyst to be easily recycled in practical applications [7,8]. Immobilization can improve some of the catalytic properties of the enzymes and also reduce their overall process costs by decreasing the amount of enzymes that are required for the processes. There are various physical and chemical methods for immobilizing enzymes, such as cross-linking, encapsulation, entrapment, covalent binding and adsorption [9]. The attachment of the enzyme to the support can be achieved chemically by covalent bonding or physical and weak bonding. The choice of the most suitable support material and the immobilization method depends largely on the type of catalytic process that will occur [10,11].

In the last decade, different cellulase immobilization techniques have been developed: covalent immobilization to chitosan [12] and monodisperse polyurea microspheres [13], immobilization of styrene/maleic anhydride copolymers [14] and alginate beads [15], chitosan-cellulase nanohybrids, entrapment in alginate gel [16] and entrapment in hybrid sol–gel matrices [17], etc. Among the immobilization methods, entrapment has the advantage of increasing the stability without direct attachment to the support matrix, thereby avoiding the possible alteration of the catalytically active conformation of the enzyme. During entrapment, an enzyme is incorporated into a membrane, a microcapsule, a fiber, or a gel network (polymer network), such as a silica sol-gel or an organic polymer. The synthesis of the polymer network in the presence of the enzyme is often required for entrapment and can be a possible source of partial inactivation [11].

The sol–gel entrapment is a remarkable method for the synthesis of silica nanoparticles and nanocomposites consisting of the preparation of a liquid “sol” (colloidal suspension of particles), its transformation into a gelatinous network (the “gel” phase) with its subsequent post-treatment (removal of the solvent) and transition to a solid oxide “xerogel” material [18]. The sol–gel procedure is carried out under mild synthesis conditions and the enzyme is not bound to the silica matrix, therefore the inactivation of the enzyme during immobilization is minimal [19].

Non-ionic surfactants, such as Tween 80 and a hydrophilic polymer PEG 20,000, have been explored as additives in the bioconversion of lignocellulosic biomass because they can increase the enzymatic hydrolysis yields by influencing the enzyme–substrate interactions, as well as enhancing the thermal stability of cellulolytic enzymes [20,21,22]. In addition, they could also be used as additives in the immobilization process to protect the enzyme and prevent the shrinkage of the matrix that is created around the enzyme, as shown in our previous studies [17,23,24].

Ionic liquids have also been proved to be efficient immobilization additives [24,25,26]. However, identifying the exact role of ionic liquids in the preparation of xerogels and the influence on the catalytic properties of the entrapped enzyme is not an easy task to do. Some advantages of ionic liquid addition during the sol–gel immobilization of the enzymes include their protection against inactivation by the released alcohol and the shrinkage of the gel during the maturation and drying step of the sol–gel immobilization process, an increased gelation time, and it having an influence on the gel structure (increase in the average pore radius and reduction in the pore size distribution) [26].

The separation of immobilized biocatalysts from the enzyme reaction mixture is a key challenge in biocatalytic processes, and the association of biomolecules with magnetic nanoparticles (MNPs) can facilitate this by using an external magnetic field, allowing the reuse of the enzyme in multiple batches and reducing the overall processing costs [27]. The development of nanobiocatalytic systems by enzyme immobilization on MNPs has received increasing attention in recent years, especially in the field of biomass conversion [27]. Cellulases have generally been immobilized by their physical adsorption or covalent binding onto various magnetic supports such as graphene oxide [28,29], nanotubes [30], porous biochar [31], chitosan [32,33], silica [34,35,36,37,38] or magnetic gold mesoporous silica nanoparticles [39].

MNPs are important in the immobilization of enzymes due to them having a large surface area and the presence of hydroxyl groups on their surface which allows the easy functionalization of them and the strong binding of the enzyme molecule [40]. Magnetic nanobiocatalysts have gained recognition due to them having multiple advantages such as stability, a low toxicity, biocompatibility, a high specific surface area, a low cost and a minimum mass transfer resistance [32].

Among the various magnetic nanoparticles that have been investigated as immobilization carriers are iron oxides (Fe_3_O_4_ and Fe_2_O_3_), alloys (CoPt_3_ and FePt), pure metals (Fe and Co) and spinel ferromagnets (MgFe_2_O_4_, MnFe_2_O_4_ and CoFe_2_O_4_). Fe_3_O_4_ nanoparticles are mainly used for the immobilization of enzymes due to their biocompatibility and non-toxicity [41]. Superparamagnetic iron oxide nanoparticles exhibit excellent recovery properties when they are the carrier materials for enzymes and they can be synthesized cost-effectively by the co-precipitation of iron salts [36].

The objectives of this research were the enhancement of the catalytic properties and operational stability of the commercial cellulase complex Cellic CTec2 by sol–gel entrapment and sol–gel entrapment combined with their deposition on magnetic particles. To our knowledge, the process of sol–gel entrapment with their deposition on magnetic particles has not been previously reported for the immobilization of Cellic CTec2 cellulase.

Another novelty of this research is the fine-tuning of the matrix structure with regard to the nature and ratio of the silane precursor mixtures, enzyme loading and additive that are used, thereby allowing the preparation of new biocatalysts exhibiting superior catalytic properties in the hydrolysis reaction of microcrystalline cellulose (Avicel PH-101). The morphology and structure of the sol–gel matrix of the nanobiocatalysts were also investigated by several characterization techniques, and a thorough correlation of it with the catalytic properties was observed (to the best of our knowledge, it has not yet been realized). The ultimate goal was the reuse of the magnetic sol–gel-entrapped biocatalyst in several batches of hydrolysis cycles with good remanent activity, thereby demonstrating its potential for large-scale applications.

These results reveal the advantages of the sol–gel entrapment of cellulases, such as the possibility of obtaining robust biocatalysts with a good thermal and pH stability, thereby allowing them to be easily recovered from the reaction medium for multiple uses.

## 2. Results and Discussion

### 2.1. Tailoring the Structure of the Sol–Gel Matrix for Efficient Cellulase Immobilization

#### 2.1.1. Influence of Nature and Molar Ratio of Silane Precursors on Cellulase Immobilization by Sol–Gel Entrapment

The immobilization of the commercial cellulase blend Cellic CTec2 by sol–gel entrapment was studied using binary and ternary mixtures of silane precursors of tetramethoxysilane (TMOS) and organically substituted trimethoxysilanes with non-hydrolyzable alkyl or aryl functional groups with a varying carbon chain length. The selection of an equimolar silane ratio was based on previous studies on lipases [24], which demonstrated the importance of TMOS for the formation of the xerogel matrix, but also the complete loss of the catalytic activity by using only TMOS as precursor due to the tight and overly compact structure of the preparate. The use of hybrid organic–inorganic matrices for the sol–gel entrapment of enzymes allows for the tailoring of the structure of the sol–gel biocatalysts, thereby leading to there being specific structural and functional properties of the entrapped enzymes. The deposition of the sol–gel-entrapped biocatalyst onto a solid support such as magnetic particles improve the mass transfer of the substrate to the catalytic center of the enzyme as a consequence of the distribution of the xerogel onto the surface of the support material. Furthermore, the use of magnetic particles as adsorbents imbues magnetic properties to the immobilized biocatalyst, thus allowing the facile separation and recycling of the enzyme.

In our study, two types of magnetic supports with high saturation magnetization properties were used, coded MP1 and MP2. The nanosized magnetic particles were synthesized by a chemical coprecipitation according to the procedure that is described in Section 4.2.2. The magnetization of the magnetic particles is slightly different as the magnetization of the nanoparticles is highly dependent on the particle size and the type of iron oxide that is present (magnetite/maghemite), as can be seen in Figure S1 in the Supplementary Materials.

The properties of the silica gel network (silane type and molar ratio, which are arranged by the increase of the C atom content), the protein *immobilization yields* and the *protein loadings* (which are expressed as mg protein per g of solid xerogel) of the immobilized biocatalysts, as well as the catalytic properties of them (the *catalytic efficiency* is expressed as the U (µmol·min^−1^·g biocatalyst^−1^) of the total sugar that is released per g of biocatalyst (sol–gel or magnetic sol–gel)) and the *specific activity* that is expressed as the U of the total sugar that is released per g of protein (µmol·min^−1^·g protein^−1^), as determined in the CMC hydrolysis, are given in Table 1.

The results depict that there is a steady decrease in the immobilization yields accomplished by the sol–gel immobilization with the increasing chain length (increasing hydrophobicity of the silane precursor molecule) of the organic function of the silanes. The immobilization yields of the enzyme in the hybrid silica matrix were usually greater than 80%, demonstrating the high effectiveness of the sol–gel entrapment process. Through using the combined method of sol–gel entrapment and the deposition of them onto magnetic particles, the immobilization yields were decreased to about 55%.

The presence of non-hydrolyzable alkyl or aryl groups in the silane precursors decreased the rate of the polycondensation reaction, thereby leading to prolonged gelling times. With an increasing C chain length of organically modified silane, we also observed a decrease in the immobilization yields; this was especially true for silanes with side chains in the organic group of the silane (such as *i*-BuTMOS) or aromatic groups (such as PhTMOS).

The catalytic efficiencies of the entrapped biocatalysts followed a different trend, their values increased with the length of the C atom chain until there were up to four C atoms in the silane precursor structure. The highest catalytic efficiency was determined for the *i*-BuTMOS:TMOS silane network at an equimolar ratio. However, the ones with longer C chains (OcTMOS) and the bulky functional groups such as an aromatic ring (PhTMOS) had a negative effect on the catalytic efficiency of the biocatalysts, suggesting that a very hydrophobic microenvironment could hinder the access of the substrate (CMC) to the active site of the enzyme. Surprisingly, the MeTMOS:TMOS silane network also led to there being very high catalytic activity of the cellulase. Interestingly, the VTMOS:TMOS silica network that contained the vinyl groups in silane demonstrated a catalytic efficiency that was far better than that of the EtTMOS:TMOS silica network with the same amount of C atoms in the organic function at similar immobilization yields and protein loadings. We attributed this to the different electronic interactions of the unsaturated ethenyl group with the enzyme molecule, compared to those of the saturated ethyl group, since the electronic interactions of the charged and polar protein side chains with the dipole moments of the substrate molecules are important for the enzyme activity [42], and the interactions with the pendant groups of the matrix could interfere in this relationship.

The catalytic efficiency of the native cellulase which was determined with the CMC assay was 36.2 U·mL^−1^**.** The enzyme blend contains approximately 70 mg of protein per mL of commercial cellulase, thus pertaining to a specific activity of 517 U·g protein^−1^. The specific activities of the sol–gel-entrapped cellulase were in the range of 101–366 U·g protein^−1^ (Table 1). With the exception of the sol–gel-containing pendant phenyl groups (SG6 sample), we observed a preservation of up to 71% of the enzyme’s specific activity after the immobilization of the MeTMOS:TMOS, PrTMOS:TMOS, and *i*-BuTMOS:TMOS silane systems. These results demonstrate that the sol–gel entrapment method is an efficient immobilization technique for cellulases.

Based on these results in the sol–gel entrapment of the Cellic CTec2 cellulase blend in binary silica networks, the next objective was to improve the immobilization yield and catalytic efficiency of the PhTMOS:TMOS silica network. We hypothesized that the lower catalytic efficiency and immobilization yields that were obtained with this binary silane mixture were due to the presence of bulky phenyl groups, thereby leading to a polymer network that was less suitable for the cellulose hydrolysis process. The fine-tuning of the sol–gel structure by switching from a binary to a ternary silane system, which contained MeTMOS or VTMOS next to PhTMOS and TMOS, could create a sol–gel matrix that is more adequate for the cellulose substrate. Such an improvement in activity by switching to ternary precursor systems has been reported for lipases [43], but it has not been investigated in the case of cellulases.

The protein immobilization yields, protein loadings of the immobilized biocatalysts, and catalytic properties (CMC assay) that were obtained using the ternary sol–gel networks with varying aromatic group contents are presented in Table 2.

The ternary mixture of the silane precursors MeTMOS:PhTMOS:TMOS and VTMOS:PhTMOS:TMOS that were in a 0.4:1.6:1 molar ratio yielded the highest catalytic efficiency of the entrapped cellulase. Introducing even small concentrations of methyl or vinyl groups into the PhTMOS:TMOS silane system led to a three-fold increase in the catalytic efficiency of the sol–gel entrapped cellulase, from 2.2 U·g^−1^ to 6.6 and 6.7 U·g^−1^, respectively. In terms of the specific activity, the ternary silane systems significantly improved the cellulase activity, especially for the magnetic biocatalysts, where a five-fold increase was observed.

Increasing the content of the vinyl group in the ternary silane network allowed for the acquiring of better immobilization yields, which were up to 90%. However, the catalytic efficiency of the cellulase remained at similar levels, even at the maximum level of protein loading (18.3 mg of protein per g of sol-gel).

In conclusion, the catalytic properties of the sol–gel-immobilized cellulase are strongly influenced by the nature and content of the non-hydrolysable substituent groups in the structure of the silane precursor. The fine-tuning of the carrier structure by using an adequate ternary precursor mixture led to a significant improvement of the catalytic properties and allowed for the further investigation of other immobilization parameters.

#### 2.1.2. Screening of Additives for Sol–Gel Biocatalysts with Improved Activity and Stability

The protection of enzymes during the sol–gel entrapment process is essential to prevent enzyme inactivation by gel shrinkage throughout the gel maturation and drying steps, or by them having an inadequate pore size, thereby resulting in slow diffusion rates. The additives that were used in the immobilization process of the enzymes not only served to improve the properties of the sol–gel matrix, but also influenced the catalytic properties of the entrapped biocatalysts. Silica gels are capable of entrapping large amounts of such additives, thus enabling the preservation or even enhancement of enzyme activity and selectivity [26]. For this reason, several compounds such as PEG (20,000 Da molecular weight), the surfactant Tween 80 [polyoxyethylene (20) sorbitan monooleate] and three ionic liquids, 1-octyl-3-methylimidazolium tetrafluoroborate (OmimBF_4_), 1-butyl-3-methylimidazolium hexafluorophosphate (BmimPF_6_) and 1-ethyl-3-methylimidazolium acetate (EmimAc) have been tested as additives in the immobilization procedure. The role of these immobilization additives was to protect the enzyme during the immobilization process and to promote a suitable porous structure of the sol–gel matrix. 

Previous studies on the potential of PEG, Tween 80 and ionic liquids to enhance the catalytic activity of enzymes [17,23,25] led us to consider them as suitable additives in the sol–gel immobilization procedure.

**Figure 1:**
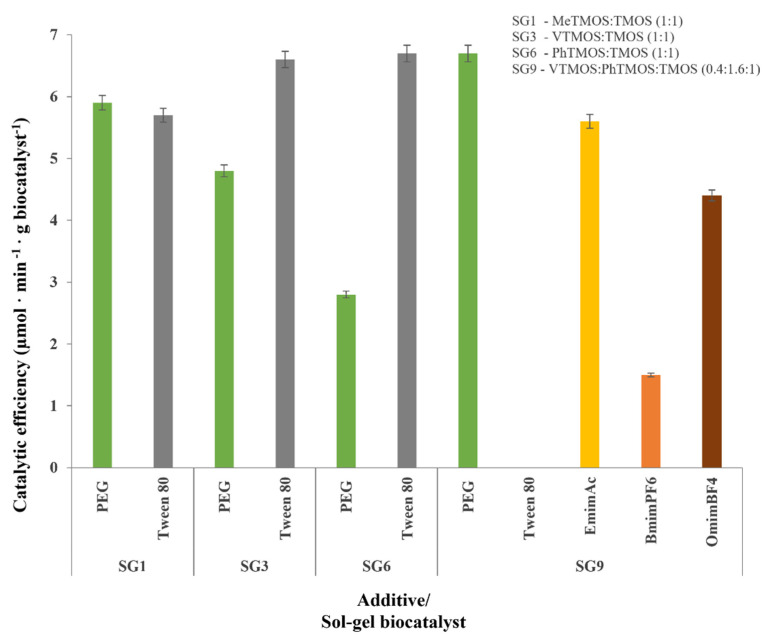
Influence of the nature of the immobilization additive on the catalytic efficiency of Cellic CTec2 cellulase that was immobilized by sol–gel entrapment with binary and ternary silane networks.

The biocatalysts that were immobilized in the binary and ternary silane networks, which are presented in Table 1 and Table 2, were obtained by using PEG 20,000 as additive. In Figure 1, these results are compared with those that were obtained using Tween 80 and the ionic liquids OmimBF_4_, BmimPF_6_ and EmimAc (only for the ternary system SG9), thus illustrating the effect of the various additives on the catalytic properties of the sol–gel entrapped enzyme.

The additives exhibited a critical influence on the catalytic efficiency of the immobilized cellulase. The addition of Tween 80 to the sol in the entrapment of the Cellic CTec2 cellulase significantly improved the catalytic efficiency of the immobilized biocatalysts, particularly for the binary silane systems with more hydrophobic non-hydrolysable functional groups such as the phenyl groups. This enhancement in the catalytic efficiency of the immobilized biocatalysts by the addition of Tween 80 was accompanied by a delay in the gelation process, and it led to the presence of immobilized biocatalysts with significantly lower protein loadings (data not shown).

Through using the binary VTMOS:TMOS silane system, the catalytic efficiency increased from 4.8 to 6.6 µmol·min^−1^·g biocatalyst^−1^, while for the PhTMOS:TMOS silane system, an increase from 2.8 to 6.7 µmol·min^−1^·g biocatalyst^−1^ (CMC assay) was observed. In case of the ternary silane system (VTMOS:PhTMOS:TMOS) that used Tween 80 as an additive, the gelation step could not be accomplished. 

Replacing the non-ionic surfactant Tween 80 with ionic liquids resulted in improved sol–gel biocatalysts being produced with increased protein loadings. The immobilization yields were above 90%, regardless of the type of ionic liquid that was used (data not shown). Since almost all of the enzyme proteins were entrapped in the sol–gel matrix, we assumed that the differences in the enzyme activity were caused by there being different inclusions of the enzyme within the porous support. Regarding the catalytic efficiency of the immobilized biocatalysts that were obtained by using ionic liquids as additives, the highest activity was observed when we were using the ionic liquid EminAc, and the catalytic efficiency increased in the series of BmimPF_6_ < OmimBF_4_ < EmimAc.

These results show that ionic liquids can be considered to be good immobilization additives because they do not interfere with the polycondensation reaction, thereby leading to improved silica gels with high protein loadings. However, the examined ionic liquids resulted in a lower catalytic efficiency of the immobilized cellulase compared to the additive PEG 20,000.

In conclusion, for the binary silane precursor system, Tween 80 was the best additive that we used. The highest value for the catalytic efficiency, which was obtained with the PhTMOS:TMOS silane precursor system and when Tween 80 was an additive, was about 6.7 µmol·min^−1^·g^−1^. Similarly, the highest catalytic efficiency, which was obtained when we used PEG as an additive in the ternary VTMOS:PhTMOS:TMOS silane precursor system (at a molar ratio of 0.4:1.6:1), was 6.7 µmol·min^−1^·g^−1^. Therefore, for the ternary silane precursor system, PEG 20,000 was the best immobilization additive that we used.

Based on these results, the ternary silane mixture VTMOS:PhTMOS:TMOS at a 0.4:1.6:1 molar ratio, in the biocatalyst SG9, is the optimal composition for the immobilization of Cellic CTec2 cellulase. Consequently, this immobilized biocatalyst SG9 was used in the subsequent experiments.

#### 2.1.3. Influence of Enzyme Loading on the Catalytic Efficiency of Immobilized Cellic CTec2 Cellulase

A key feature of sol–gel structures is the possibility of combining high protein loadings with a minimal diffusion limitation [19].

One of the main advantages of sol–gel immobilization is the ability to entrap a larger amount of enzyme proteins in comparison to other immobilization methods. However, the presence of the entrapped enzyme in the sol–gel matrix that is above a certain protein loading can impede substrate diffusion to the active center of the enzyme, thereby leading to a lower catalytic efficiency of the immobilized biocatalyst. This is especially true for cellulases since their natural substrates (cellulose) are high-molecular mass compounds. This aspect was highlighted in this study.

The effect of protein loading on the catalytic efficiency of the biocatalysts was studied by evaluating the catalytic efficiency of the immobilized cellulases at three initially added protein amounts (1.66, 3.33 and 5.00 mg of protein per mmol of silanes).

**Figure 2:**
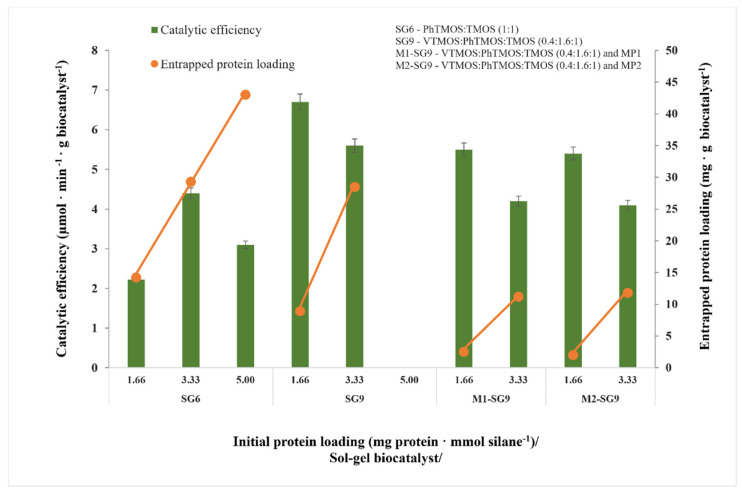
Catalytic properties of immobilized Cellic CTec2 cellulase which was obtained by its entrapment in sol–gel and magnetic sol–gel at different enzyme loadings.

The catalytic efficiencies (DNS assay) of the immobilized biocatalysts, obtained by entrapment in sol–gel using a PhTMOS:TMOS binary silane precursor system at a 1:1 molar ratio (SG6) and sol–gel (SG9) and magnetic sol–gel (M1-SG9 and M2-SG9) using a VTMOS:PhTMOS:TMOS ternary silane precursor system at a 0.4:1.6:1 molar ratio and different protein loadings, are presented in Figure 2.

As highlighted in Section 2.1.1, the catalytic efficiency for the binary sol–gel biocatalyst SG6 at an initial protein loading of 1.66 mg·mmol silane^−1^ was 2.2 µmol·min^−1^·g biocatalyst^−1^. Increasing the initial protein amount led to a proportional increase in the entrapped protein loading. At 3.33 mg·mmol silane^−1^, we observed a two-fold increase in the catalytic efficiency, while higher amounts resulted in a slight decrease in the catalytic efficiency.

For the biocatalyst that was obtained with a ternary silane system, with a protein dosing of 1.66 mg·mmol silane^−1^, the VTMOS:PhTMOS:TMOS ternary silane system led to an entrapped protein amount of 8.9 mg protein per g sol–gel and the highest catalytic efficiency value (6.7 µmol·min^−1^·g biocatalyst^−1^). A two-fold increase in the initial protein amount (3.33 mg·mmol silane^−1^) lead to a protein loading of 28.5 mg protein per g sol–gel, but it lead to a small decrease in the catalytic efficiency of only 5.6 µmol·min^−1^·g biocatalyst^−1^. Thus, increasing further the amount of protein-added (5.00 mg protein per mmol silane) gelation could not be accomplished for the biocatalysts within the ternary silane system.

For the sol–gels with ternary silane systems that were adsorbed onto the magnetic particles (M1-SG9 and M2-SG9), the calculated protein loadings were considerably lower due to the higher mass fraction of the immobilized biocatalyst with a non-catalytic function. At a protein loading of 1.66 mg·mmol silane^−1^, the magnetic sol–gel biocatalysts that are presented in this work have similar catalytic efficiencies with the sol–gel-immobilized biocatalyst (approximately 5.5 µmol·min^−1^·g biocatalyst^−1^), but with significantly lower protein loadings (2.0–2.5 mg protein·g biocatalyst ^−1^). By increasing the protein loading to 3.33 mg·mmol silane^−1^, up to a six-fold increase in the entrapped protein loading was observed, which correlated with a slight decrease in the catalytic efficiency.

The protein content in the immobilization mixture has an important influence as concentrations above a certain limit can impede the gel formation, whereas low concentrations may lead to there being reduced immobilization yields.

Based on these results, a protein loading of 3.33 mg·mmol silane^−1^ for the binary and ternary silane systems sol–gels and the magnetic sol–gel that were obtained can be considered optimal for the immobilization of Cellic CTec2 cellulase.

### 2.2. Morphological and Structural Characterization of Immobilized Cellulase

#### 2.2.1. Scanning Electron Microscopy (SEM)

**Figure 3:**
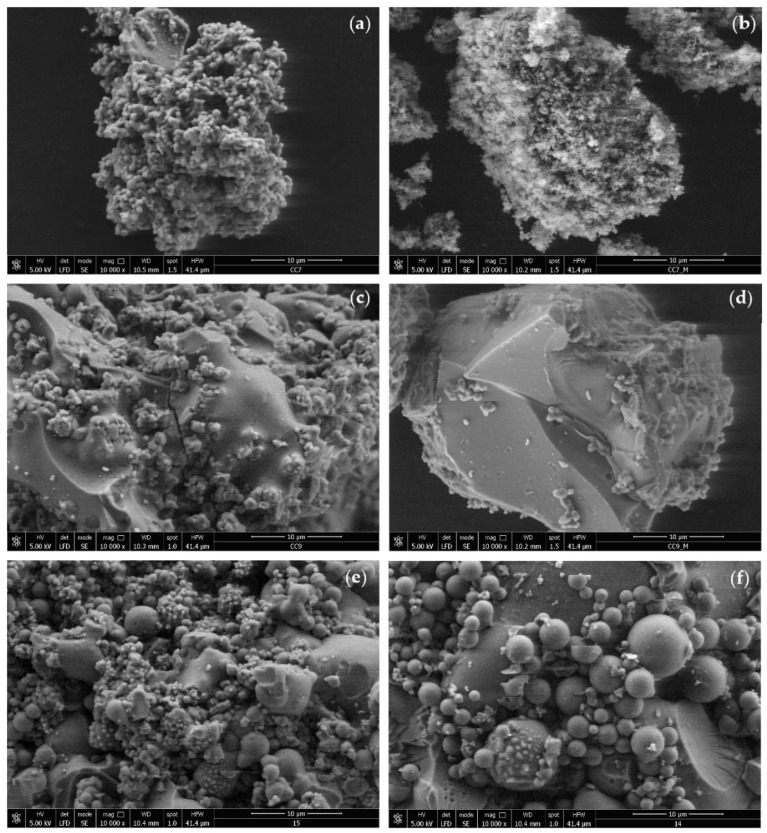
SEM micrographs at 10,000 × magnification of the sol–gel entrapped cellulase biocatalysts and the blank sol–gel matrix: (**a**) SG6—PhTMOS:TMOS (1:1); (**b**) Blank-SG6—PhTMOS:TMOS (1:1); (**c**) SG9—VTMOS:PhTMOS:TMOS (0.4:1.6:1); (**d**) Blank-SG9—VTMOS:PhTMOS:TMOS (0.4:1.6:1); (**e**) SG10—VTMOS: PhTMOS:TMOS (1:1:1); (**f**) SG11—VTMOS:PhTMOS:TMOS (1.6:0.4:1).

Through scanning electron microscopy (SEM), important characteristics of the morphology of the immobilized biocatalysts can be highlighted as they provide useful information about the microstructure, porosity, and texture of the studied material, and these are details that can be correlated with their catalytic efficiency. A SEM characterization study was performed to analyze the effect of different precursor silanes and the immobilization method that was used on the morphology of the sol–gel material with entrapped cellulase. The SEM images are shown in Figure 3 at 10,000× magnification.

The SEM image for the entrapped biocatalyst (SG6) that was obtained by the use of the direct sol–gel method with a binary system of precursor silanes PhTMOS:TMOS at a molar ratio of 1:1 (Figure 3a) shows a porous structure containing particles <2 µm (even sub-micrometric) in a very crowded structure, and the SEM image of the control sol–gel matrix (Figure 3b) is not much different from that of the immobilized biocatalyst.

The entrapped biocatalyst (SG9) that was obtained with the ternary silane precursor system VTMOS:PhTMOS:TMOS at a molar ratio of 0.4:1.6:1, with a higher content of phenyl groups, shows (Figure 3c) a porous structure with smooth areas and tiny particles that are generally less than 4 µm, which firmly adhered to the smooth surfaces of the large particles; this is a structure that is correlated with the good catalytic efficiency values that were obtained in the CMC hydrolysis reaction. In contrast, the structure of the control gel is completely different, with it showing a block-like morphology with almost completely smooth surfaces and sharp edges.

With the decrease in the concentration of the phenyl groups in the sol–gel matrix and the increase in the content of the vinyl groups in the entrapped biocatalysts, their SEM images show that there are more compact structures, with an increase in the size of the spherical particles that end up measuring 5–10 µm and are found on the compact surface of the matrix (Figure 3e,f). These structures are correlated with a slightly lower catalytic efficiency, probably due to the fact that sol–gel matrices become too dense and compact, thereby causing a limitation in the transfer of the corresponding mass of the reactant and the product [24,44].

**Figure 4:**
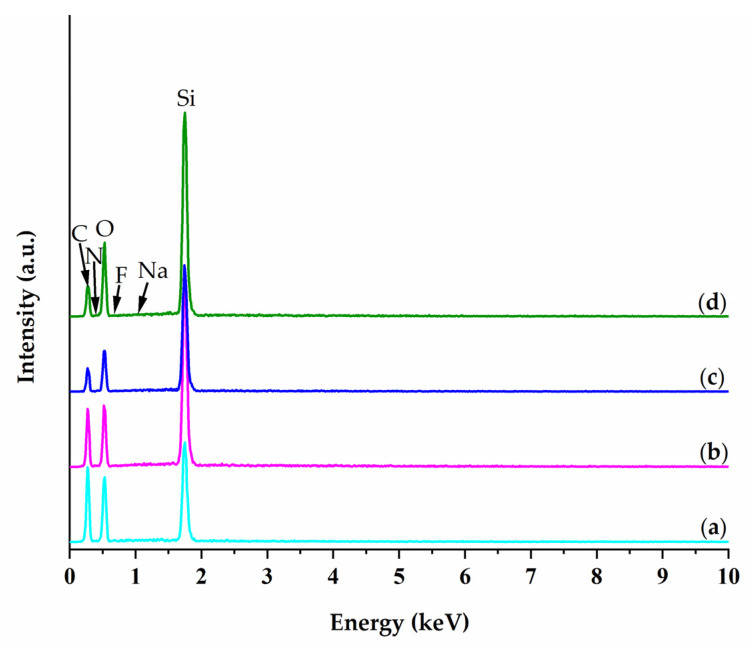
EDS spectra of the investigated biocatalysts: (**a**) SG6—PhTMOS:TMOS (1:1); (**b**) SG9—VTMOS:PhTMOS:TMOS (0.4:1.6:1); (**c**) SG10—VTMOS:PhTMOS:TMOS (1:1:1); (**d**) SG11—VTMOS:PhTMOS:TMOS (1.6:0.4:1).

Figure 4 shows the EDS spectra of the investigated materials, and it can be noticed that the main peaks are attributed to C, O and Si, which is normal for these types of materials and it is in concordance with our previous studies [25].

In addition to these peaks with a higher intensity, N, F and Na were detected as well. N is present in small amounts, and it comes from the protein that is confined in the matrix, while traces of F and Na elements were detected with a wt. % that is less than 1%, thus indicating that a small part of the NaF catalyst was not eliminated during the gel-washing step.

The relative ratio of C/Si (*w*/*w*) in the silane precursors that were employed for the studied immobilized biocatalysts decreased from 6.13 to 2.33, and this is corelated with the decrease in the phenyl group in the sol–gel matrix. All the peaks are revealed by the quantification results that are presented in Table 3.

#### 2.2.2. Fluorescence Microscopy (FM)

**Figure 5:**
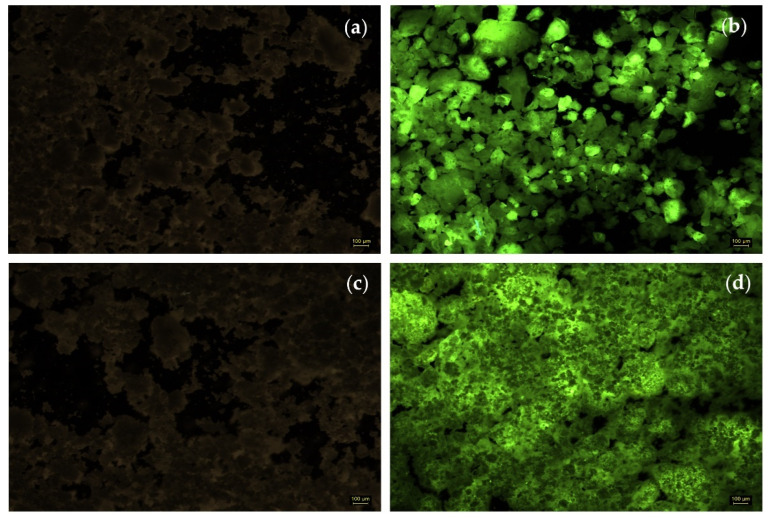
Fluorescent images of blank sol–gel matrices (without enzymes) and the sol–gel matrices containing FITC-labelled cellulase: (**a**) Blank-SG6 matrix—PhTMOS:TMOS (1:1); (**b**) FITC-SG6—PhTMOS:TMOS (1:1); (**c**) Blank-SG9 matrix—VTMOS:PhTMOS:TMOS (0.4:1.6:1); (**d**) FITC-SG9—VTMOS:PhTMOS:TMOS (0.4:1.6:1).

Fluorescence microscopic imaging provides important information on the distribution of the immobilized enzyme within the sol–gel matrix, which is essential for the accessibility of the enzyme by the substrate molecules. Because commercial Cellic CTec2 cellulase does not have natural fluorescence, it was necessary to label it with a fluorochrome compound, fluorescein isothiocyanate (FITC). The labelling was performed according to the method that is described in the PIERCE EZ-LabelTM FITC Labeling Kit, then the immobilization was carried out using the same protocol as for unlabeled cellulase’s entrapment in the sol–gel (0.150 mL suspension containing cellulase that was bound to the fluorescein solution was immobilized by its entrapment in the sol–gel using the precursor silanes PhTMOS:TMOS at a 1:1 molar ratio and VTMOS:PhTMOS:TMOS at a 0.4:1.6:1 molar ratio). For comparison, a control sol–gel matrix (without an enzyme) was used for each immobilized biocatalyst that was labeled with FITC, as shown in Figure 5a,c.

The images that were obtained by us applying fluorescence microscopy to the cellulase–FITC complex that was immobilized in the two types of sol–gel matrices are shown in Figure 5b for the binary system of precursor silanes PhTMOS:TMOS at a 1:1 molar ratio and in Figure 5d for the ternary silane system VTMOS:PhTMOS:TMOS at a 0.4:1.6:1 molar ratio. Because only the FITC-labelled biocatalysts showed fluorescence, the surface profile of the active immobilized cellulases demonstrates that the enzyme is distributed both on the surface and inside of the xerogel; in both cases, the labelled cellulases are found throughout the matrix, suggesting that there is a uniform distribution of the enzyme in the preparations obtained by the sol–gel entrapment technique. At the same time, in the case of the sol–gel matrix that was obtained with a binary system of precursor silanes PhTMOS:TMOS at a 1:1 molar ratio, which is shown in Figure 5b, a different fluorescence is observed than that which was obtained with the ternary system VTMOS:PhTMOS:TMOS at a 0.4:1.6:1 molar ratio; the fluorescence image shows that there is the presence of more compact blocks that keep the enzyme tight, which is a structure that can be correlated with the lower enzymatic efficiency of the biocatalyst.

#### 2.2.3. FT-IR Spectroscopy

**Figure 6:**
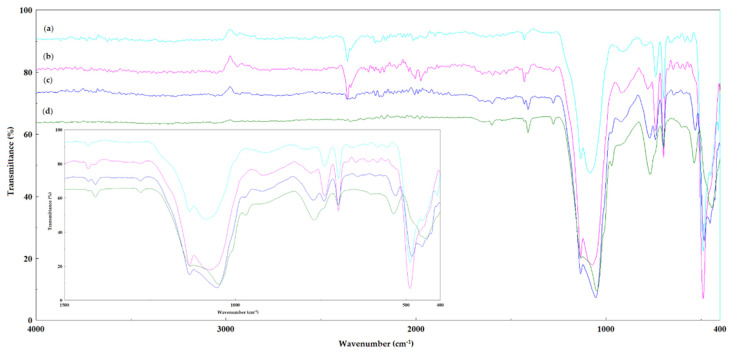
FT-IR spectra of cellulase immobilized biocatalysts, obtained by the sol–gel entrapment method: (**a**) SG6—PhTMOS:TMOS (1:1); (**b**) SG9—VTMOS:PhTMOS:TMOS (0.4:1.6:1); (**c**) SG10—VTMOS:PhTMOS:TMOS (1:1:1); (**d**) SG11—VTMOS:PhTMOS:TMOS (1.6:0.4:1). Inset: enlarged picture of the 400–1500 cm^−1^ region.

FT-IR spectroscopy is a technique that is often used to characterize the structure of the sol–gel type materials. The FT-IR spectra confirmed the presence of alkyl and aryl functional groups of the precursor silanes in the sol–gel matrix (Figure 6), but the FTIR spectroscopic analysis could not locate the cellulase because the band of the amide group was covered by other vibrations of the structure sol–gel matrices.

All of the immobilized biocatalysts showed characteristic bands of silica compounds that were formed by the hydrolysis/condensation of the silane precursor groups that were used: the 1085, 1076, 1056 and 1049 cm^−1^ bands were attributed to the Si–O–Si bonds (1090–1030 cm^−1^); the 786, 777 and 767 cm^−1^ bands were specific to Si-CH_3_ bonds (860–760 cm^−1^) [45]. The intense Si-OH bands between 3700–3200 cm^−1^ are missing, which proves that almost all of the OH groups were involved in the sol–gel matrix, thereby confirming the relationship between the condensation and contraction reactions of different types of precursor silanes. At the same time, the absence of the residual Si-OH groups also explains the hydrophobicity of the sol–gel matrix, as has been previously shown [24].

The presence of the phenyl groups in the immobilized biocatalysts (Figure 6) is demonstrated by the specific bands of the CH group from the aromatic nucleus of benzene and the vibration of the aromatic skeleton at 734–740 cm^−1^ and 696–698 cm^−1^, respectively. These bands are best observed in the spectra of the immobilized biocatalysts where the phenyl groups are in higher concentration (SG6) with PhTMOS:TMOS at a 1:1 molar ratio, Figure 6a, and (SG9) with VTMOS:PhTMOS:TMOS at a 0.4:1.6:1 molar ratio, Figure 6b. The bands at 1409 cm^−1^ and 968 cm^−1^ can be assigned to the ν_s_ and δ_CH2_ of the Si–CH=CH_2_ bonds (1410–1390 cm^−1^; 960–980 cm^−1^), and the intensities are higher in the biocatalyst (SG11), and this corresponds to there being a higher concentration of vinyl groups in the sol–gel matrix.

These results confirm that all of the precursors were included in the sol–gel matrix; the presence of these functional groups is essential for the activity and operational stability of the entrapped enzyme.

**Figure 7:**
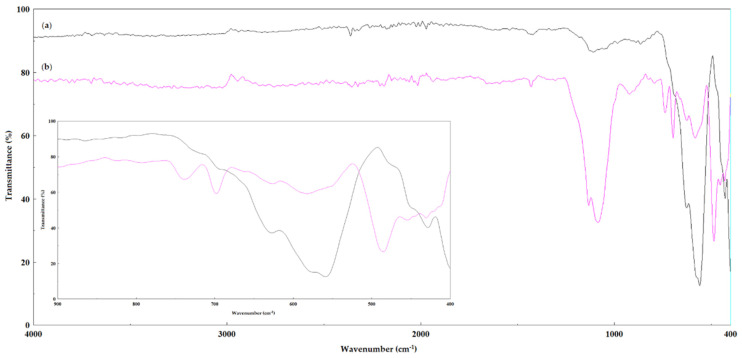
FT-IR spectra of: (**a**) magnetic support MP1 and (**b**) cellulase biocatalyst (M1-SG9), which were obtained by the magnetic sol–gel immobilization method with silane precursors VTMOS: PhTMOS:TMOS (0.4:1.6:1) and MP1. Inset: enlarged picture of the 400–900 cm^−1^ region, showing the presence of the specific absorption bands for the magnetite/maghemite mixture.

Regarding the biocatalysts that were obtained by the double immobilization (sol–gel magnetic), the FT-IR spectra that were obtained are different because the specific bands of the magnetic support also appeared (Figure 7).

Therefore, the FT-IR spectrum of the magnetic support MP1, Figure 7a, shows that there were intense bands at 559 cm^−1^ and 400 cm^−1^ that are specific to magnetite (Fe_3_O_4_, 570 cm^−1^ and 400 cm^−1^) as well as weaker bands at 628 cm^−1^ and 430 cm^−1^ that are specific to maghemite (γ-Fe_2_O_3_ 630–660 cm^−1^), which shows that this support is a mixture of magnetite/maghemite. In the case of the immobilized biocatalyst M1-SG9 that was obtained by the sol–gel entrapment method which was combined with its adsorption on the magnetic support MP1, with a ternary system of precursor silanes (PhTMOS:VTMOS:TMOS at a 1.6:0.4:1 molar ratio), Figure 7b, the specific bands of the magnetic support, as well as those of the sol–gel matrix, have been found.

The FT-IR spectrum of the magnetic support MP2, Figure S2a (Supplementary Materials), shows that there were bands of medium intensity at 634 cm^−1^, 582 cm^−1^ and 433 cm^−1^ that are specific to maghemite γ-Fe_2_O_3_, as well as very weak bands at 565 cm^−1^ and 410 cm^−1^ that are specific to Fe_3_O_4_ magnetite, thus proving that it is predominantly maghemite, which contributes to its lower magnetization value when it is compared to MP1.

#### 2.2.4. Thermal Analysis (TGA/DTA) 

**Figure 8:**
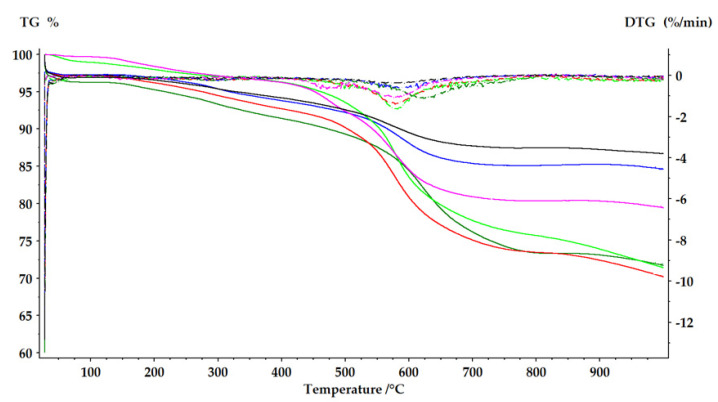
Thermograms showing weight loss (TG, continuous line) and its derivative (DTG, dotted lines) for the: biocatalyst SG6—PhTMOS:TMOS (1:1), (dark green); Blank-SG6 matrix—PhTMOS:TMOS (1:1), (light green); biocatalyst SG9—VTMOS:PhTMOS:TMOS (0.4:1.6:1), (red); Blank-SG9 matrix—VTMOS:PhTMOS:TMOS (0.4:1.6:1), (purple); biocatalyst SG10—VTMOS:PhTMOS:TMOS (1:1:1), (blue) and SG11—VTMOS:PhTMOS:TMOS (1.6:0.4:1), (black).

A thermogravimetric analysis (TGA) provides important information about the temperature range in which the samples undergo a major conformational change by observing the mass loss profile as a function of temperature. The mass loss curves of the biocatalysts that were immobilized by the sol–gel entrapment method were divided into three regions (Figure 8 and Table 4), as seen in our previous studies [24].

Region I is related to the loss of water and some volatile organic molecules, which goes up to a temperature of 280 °C. Region II, which is in the range of 280–530 °C, is associated with the loss of organic constituents (C, H, O and N), volatiles that were present or were formed at the beginning of the organic decomposition, including cellulase decomposition. In region III, the mass loss is associated with the dehydroxylation and carbonization reactions of the organic compounds. Above 850 °C, thermal stability may be reached, or the material may be completely disintegrated.

In the first interval, the mass loss between 2.1–3.8% was due to the evaporation of water and perhaps a small amount of 2-propanol or other volatile compounds that remained in the preparation at the end of the drying period. It was not surprising that the immobilized biocatalysts that contain the entrapped cellulase had a mass loss that is about 0.4–1% higher than the cellulase-free sol–gel matrix had, which used as reference, since the cellulase was introduced as such in liquid form into the immobilization mixture and it remained in a hydrated state at the end of the process. The small amount of water that is hydrogen-bonded to the hydrophilic amino acid residues in the enzyme structure is essential to maintain the conformation of the active site. The second and third regions are the most important for the thermal analysis profiles of the immobilized biocatalyst; the difference of 0.11–0.13% between the mass loss of the immobilized biocatalysts (PhTMOS:TMOS at a 1:1 molar ratio), respectively (VTMOS:PhTMOS:TMOS at a 0.4:1.6:1 molar ratio), and the reference control gels, in the range of 280–530 °C can be attributed to the entrapped protein.

The decrease in the mass loss that was observed in the immobilized biocatalysts obtained with a ternary system of silane precursors with the decrease of phenyl groups, respectively, with the increase of vinyl groups in the sol-gel matrix, is correlated, as seen, with a decrease in the catalytic efficiency of these biocatalysts, probably because of the compaction of the matrix network, as was also noticed by SEM scanning electron microscopy.

The DTG curves, Figure 8, show that the highest value of the thermal decomposition rate was at high temperatures of around 600 °C, which indicates that there was a very good protection against temperature rise after the immobilization. These conclusions were later confirmed by the preservation of the cellulase activity at high temperatures, which was also demonstrated by the thermal stability study.

The organic functional groups that were covalently bound in the matrix structure were almost completely decomposed up to 800 °C in all of the immobilized biocatalysts that were tested. As can be seen in Table 4, the total amount of mass that was lost was the highest (about 28%) in the case of the immobilized biocatalysts with the highest concentration of phenyl groups, and this loss decreased as the number of vinyl groups in the matrix increased.

### 2.3. Tuning the Catalytic Efficiency of the Sol–Gel-Entrapped Cellulase for the Hydrolysis of Cellulose

#### 2.3.1. Time Course of the Enzymatic Hydrolysis of Microcrystalline Cellulose

**Figure 9:**
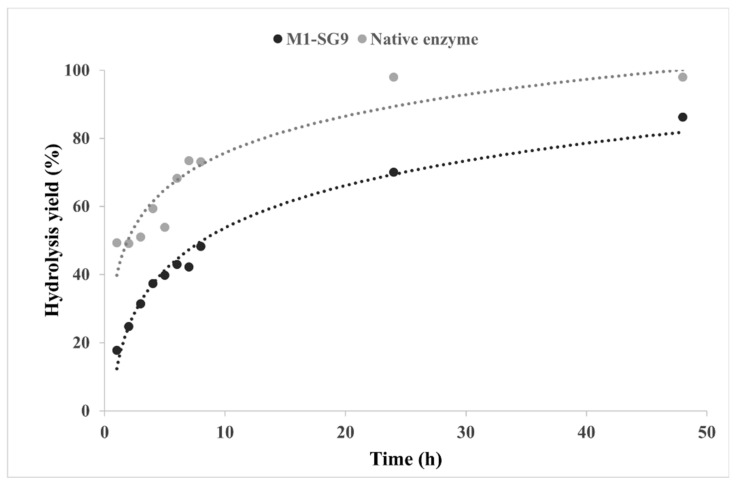
Time course of the enzymatic hydrolysis reaction of Avicel PH-101 cellulose that was catalyzed by native (grey) and M1-SG9 magnetic sol–gel-immobilized cellulase (black), which was obtained by using the silane network VTMOS:PhTMOS:TMOS (0.4:1.6:1) and magnetic particles MP1 (dotted lines represent fitted curves).

The catalytic efficiency of the sol–gel-entrapped cellulase was also investigated in the hydrolysis of the microcrystalline cellulose (Avicel PH-101) substrate. The DNS assay of the total reducing sugars has been used at different time intervals, which ranged up to 48 h, to establish the optimum time of the hydrolysis reaction of the native and the magnetic sol–gel-immobilized cellulase M1-SG9 with VTMOS:PhTMOS:TMOS at a molar ratio of 0.4:1.6:1 which was adsorbed onto MP1. The time course of the hydrolysis reactions is shown in Figure 9.

Comparing the catalytic efficiency of the native and immobilized enzymes is a difficult task because, during the immobilization, the enzyme was dispersed in the sol–gel matrix, and the amount of entrapped proteins can only be determined indirectly. The native cellulase was added to the reaction medium at a concentration of 6 mL·L^−1^ (approximately 0.4 mg protein per mL acetate buffer), while for the (solid) immobilized cellulase, about 10 g·L^−1^ sol–gel was added, therefore corresponding to only 0.1 mg of protein per mL of the buffer.

We observed a steady increase in the hydrolysis yield of Avicel PH-101 as a function of time for both biocatalysts. In the case of the native cellulase, almost a complete conversion of the substrate was achieved after 24 h. The hydrolysis reaction that was catalyzed by the magnetic sol–gel-entrapped cellulase followed a similar trend, though with lower yields, after 24 h, and this reached 86% after 48 h of the reaction.

Compared to the native cellulase, the immobilized biocatalyst yielded a lower hydrolysis rate, but the immobilization confers numerous other advantages, such as an enhanced stability and reusability, as is shown in Section 2.4 and Section 2.5. The lower hydrolysis yield that was obtained for the sol–gel-entrapped biocatalysts is probably due to the reduced diffusion of the substrate in the sol–gel matrix. Further hydrolysis experiments were performed at 24 h of reaction time.

#### 2.3.2. Influence of the Immobilization Parameters on the Enzymatic Hydrolysis of Microcrystalline Cellulose

The influence of the nature and molar ratio of the silane precursors on the catalytic efficiency of the sol–gel-immobilized cellulase was explored using the reference substrate CMC, and this is discussed in Section 2.1.1. The same immobilized biocatalysts were also studied as catalysts for the hydrolysis of Avicel 101 microcrystalline cellulose. Sugar productivity, as it is defined in literature [46], was considered to be an appropriate parameter to express the real catalytic efficiency of the biocatalysts in the cellulose hydrolysis process.

**Figure 10:**
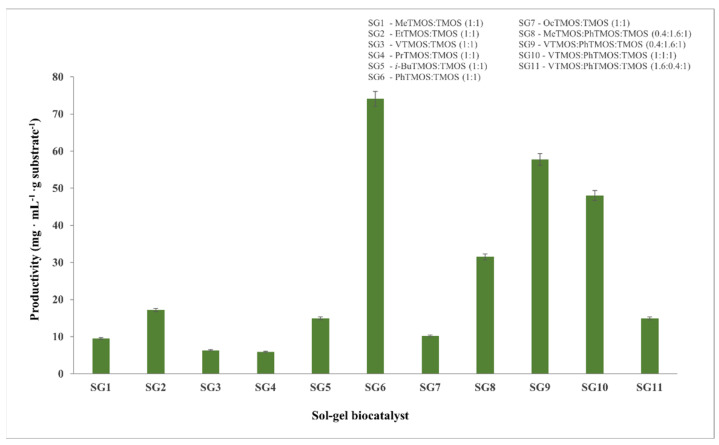
Enzymatic hydrolysis of microcrystalline cellulose Avicel-PH101 that was catalyzed by immobilized biocatalysts which were obtained by their entrapment in sol–gel, at 24 h reaction time and 50 °C.

As shown in Figure 10, the immobilization of Cellic CTec2 cellulase in the sol–gels that were obtained from the binary silane systems using alkyl-functionalized silanes resulted in low hydrolysis yields and sugar productivity values that were less than 20 mg of sugar per mL of the reaction mixture. The highest sugar productivity value (corresponding to 40% Avicel hydrolysis yield in 24 h and a sugar productivity of approximately 75 mg·mL^−1^) was obtained for the PhTMOS:TMOS system (SG6 biocatalyst). As concerns the ternary silane precursor systems, the best results were obtained for the sol–gel matrix with the highest content of the phenyl groups (SG9 biocatalyst, obtained with VTMOS:PhTMOS:TMOS at a 0.4:1.6:1 molar ratio). Increasing the vinyl group content in the tertiary silane precursor system resulted in a steady decrease of the catalytic efficiency, which was observed. Obviously, the presence of bulky phenyl groups in the matrix in an optimized amount is favorable for cellulose hydrolysis.

#### 2.3.3. Influence of Enzyme Loading on the Enzymatic Hydrolysis of Microcrystalline Cellulose

The enzymatic hydrolysis reaction of Avicel PH-101, which was catalyzed by the best performing immobilized biocatalysts which were obtained by their entrapment in sol–gel and magnetic sol–gel (presented in Table 5), was also studied at different protein loadings by means of observing their sugar productivity (mg·mL^−1^·g substrate^−1^).

In the case of the binary silane system, the productivity increased only slightly with the increasing of the protein loading, which was up to 111 mg/mL. For the sol–gel-entrapped cellulase using a ternary silane system, higher protein loadings were limited by the gelation difficulties, as is discussed earlier in the paper. The ternary system without magnetic particles (VTMOS:PhTMOS:TMOS at a 0.4:1.6:1 molar ratio) led to comparable sugar productivities as for the binary system at lower entrapped enzyme amounts. A sugar productivity value of 108 mg·mL^−1^·g substrate^−1^ was obtained at a protein loading of 28.5 mg·g biocatalyst^−1^, compared to a productivity of 111 mg·mL^−1^·g substrate^−1^ at the maximum protein loading of 43.0 mg·g biocatalyst^−1^ for the binary silane system.

By using the ternary magnetic sol–gel systems, higher productivity values were obtained with significantly lower enzyme contents of the magnetic nanobiocatalysts. The deposition on the magnetic particles probably leads to an improved mass transfer and has a positive effect on Avicel hydrolysis. Although the immobilization yield of the enzyme is lower, the biocatalyst is more efficiently disposed in the matrix, and the sugar productivity increases. Therefore, this magnetic sol–gel biocatalyst M1-SG9 can be considered to be the best option for further studies. 

### 2.4. Effect of Immobilization on the Main Parameters of the Cellulose Hydrolysis Reaction

#### 2.4.1. Influence of Temperature on the Catalytic Performance of the Immobilized Biocatalysts

**Figure 11:**
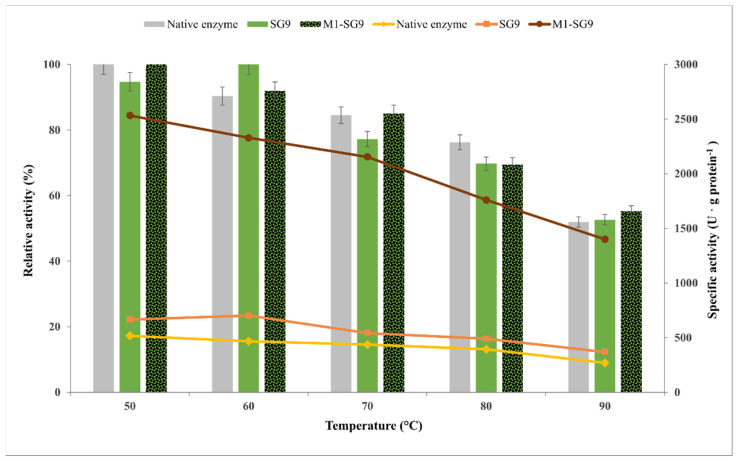
Influence of temperature on the catalytic efficiency of native and immobilized Cellic CTec2 cellulase by entrapment in sol–gel (SG9) and magnetic sol–gel (M1-SG9) with VTMOS:PhTMOS:TMOS (0.4:1.6:1) in the hydrolysis of CMC at 30 min reaction time and different temperatures in the range 50–90 °C.

Thermal stability is a key requirement for biocatalysts that are intended for use in industrial processes; thus, the development of thermostable biocatalysts through enzyme immobilization is essential. The influence of temperature on the catalytic efficiency of the native and sol–gel-immobilized biocatalysts has been studied for both of the sol–gel biocatalysts that were obtained with the ternary silane network VTMOS:PhTMOS:TMOS at a 0.4:1.6:1 molar ratio without (SG9) or with deposition on the magnetic particles MP1 (M1-SG9). The model reaction of CMC hydrolysis was carried out at different temperatures in the range of 50–90 °C. The relative activities of the immobilized biocatalysts which are compared to those of the native cellulase are shown in Figure 11.

The maximum catalytic efficiency of the native cellulase was observed at 50 °C, which was followed by a steady loss of activity at increasing temperatures, with there being about 50% at 90 °C. With respect to sol–gel-entrapped biocatalyst (SG9), we observed an increase in the temperature-related activity of the enzyme up to 60 °C; above this temperature, there was a slow decrease in the catalytic activity to 53%. An almost two-fold increase in the specific activity was determined for the sol–gel biocatalyst (702 U·g protein^−1^) at this temperature (native enzyme 467 U·g protein^−1^). The elevated temperature proved to be beneficial for the catalytic efficiency of the sol–gel biocatalyst probably because of improved substrate diffusion to the entrapped enzyme.

In the case of the magnetic biocatalyst (M1-SG9), the highest catalytic efficiency was obtained at 50 °C. A five-fold increase in the specific activity was determined for the magnetic sol–gel biocatalyst (2533 U·g protein^−1^) at this temperature (517 U·g protein^−1^ for the native enzyme). It can be noticed that the thermal inactivation at increasing temperatures was lower for the magnetic sol–gel biocatalyst. In the studied temperature range, this biocatalyst maintained the highest relative activity that was about 55% at 90 °C. Other research groups also reported that there were unchanged optimal temperature values after the immobilization or an extension of the temperature stability range [29,47,48,49].

Keeping in mind the industrial applications of immobilized enzymes, thermal stability is one of the important parameters to be considered; therefore, the magnetic sol–gel biocatalyst demonstrated promising potential in this field with the added benefit of its easy recovery by applying a magnetic field.

#### 2.4.2. Influence of the pH of the Reaction Medium on the Catalytic Performance of Sol–Gel-Immobilized Cellulase

**Figure 12:**
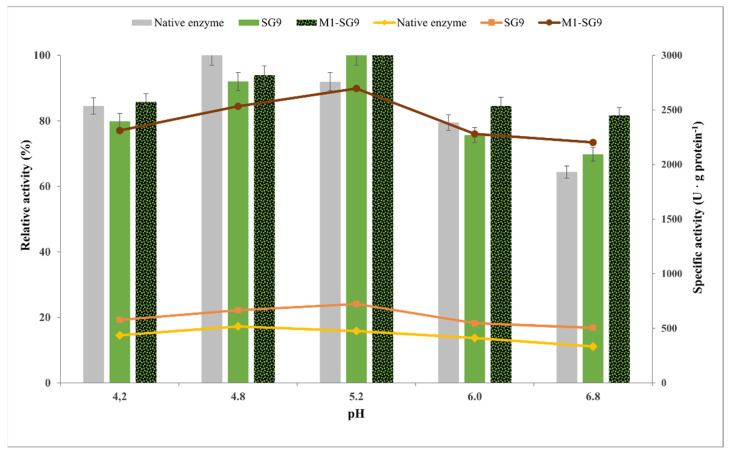
Influence of pH on the catalytic efficiency of native and immobilized Cellic CTec2 cellulase by entrapment in sol–gel (SG9) and magnetic sol–gel (M1-SG9) with VTMOS:PhTMOS:TMOS (0.4:1.6:1) in the hydrolysis of CMC at 30 min reaction time and 50 °C.

Enzymes are generally stable only at a well-defined pH range; cellulases particularly favor more acidic environments, which is why most activity assays are carried out at a pH of 4.8–5.0. The influence of pH on the catalytic performance of the native and immobilized biocatalysts (sol–gel SG9 and magnetic sol–gel M1-SG9), with the ternary silane network VTMOS:PhTMOS:TMOS at a molar ratio of 0.4:1.6:1, was studied in the range of 4.2–6.8. The relative and specific activity of the native and immobilized biocatalysts are given in Figure 12.

In the studied pH range, the native cellulase had the highest catalytic efficiency at a pH of 4.8, while the sol–gel and magnetic sol–gel-immobilized biocatalysts were most efficient at a pH of 5.2. Other research groups reported either a slight decrease or unchanged optimal pH values after immobilization [29,49,50].

An almost two-fold increase in specific activity was determined for the sol–gel biocatalyst (723 U·g protein^−1^) and a six-fold increase for the magnetic sol–gel biocatalyst (2696 U·g protein^−1^) at this pH value (native enzyme only 476 U·g protein^−1^). The immobilized biocatalysts were relatively more stable in a broader pH range when they were compared to the native cellulase. The enhancement of the pH stability of the cellulase towards a more basic media after the immobilization is due to the increased rigidity and protection of the enzyme by the sol–gel matrix against conformational changes that can be induced by pH variations, as was also seen in our previous study [17].

### 2.5. Operational Stability of the Immobilized Cellulase

Enhancing the recyclability of cellulases is a promising approach to reduce the high cost of the enzymatic hydrolysis of lignocellulosic biomass to fermentable sugars/glucose.

**Figure 13:**
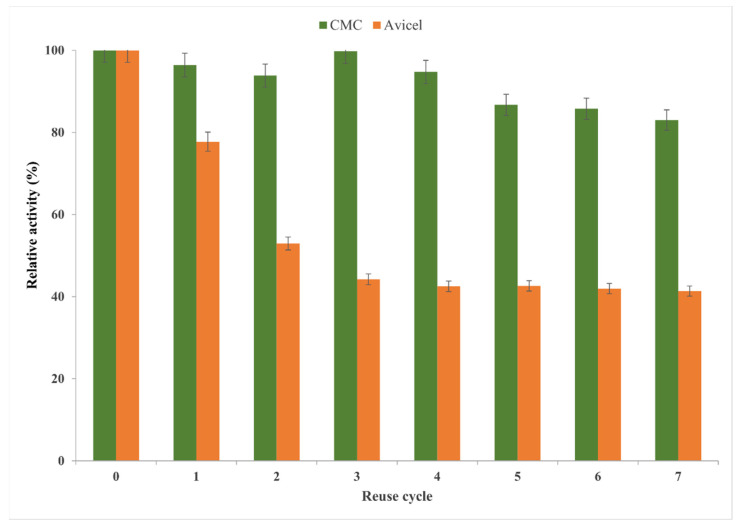
Operational stability of Cellic CTec2 cellulase that was immobilized by its entrapment in magnetic sol–gel (M1-SG9) with VTMOS:PhTMOS:TMOS (0.4:1.6:1) in the hydrolysis of CMC at 30 min reaction time at 50 °C, and Avicel PH-101 at 24 h reaction time at 50 °C.

One of the main objectives of this study was the recovery and reuse of the immobilized biocatalysts, as this can reduce the process costs. Generally, enzymes are reused until their activity reaches below 25% of their initial value. The operational stability in seven consecutive reaction cycles (Figure 13) of the magnetic sol–gel-entrapped biocatalyst M1-SG9 was evaluated in the hydrolysis of CMC at 50 °C, with a pH of 4.8, and after 30 min of reaction time in comparison with the hydrolysis of the microcrystalline cellulose Avicel PH101 at 50 °C, with a pH of 4.8, and after 24 h of reaction time.

The magnetic sol–gel biocatalyst retained more than 80% of its activity after seven reaction cycles of the hydrolysis of CMC and only 40% of its activity after seven hydrolysis cycles in comparison to it’s first use in the hydrolysis of Avicel PH-101.

The maximum glucose productivity that was obtained in the decomposition of CMC at 50 °C and at a pH of 4.8 was 171 mg·mL^−1^·g^−1^ of CMC, which is similar to the results that were reported by Tan et al. (162 mg·g^−1^ of CMC) using cellulase that was immobilized inside a hollow magnetic structure [49], and Zanuso et al. (168 mg·g^−1^ of CMC) who used cellulase that was bound to magnetic nanoparticles that were coated with chitosan [33]. Zanuso et al. found that there was a 45% relative activity retention after 13 reuse cycles for the magnetic biocatalyst, while Asar et al. [29] reported that there was a 49% retention of activity after eight reuse cycles for the cellulase that was immobilized on chitosan-modified Fe_3_O_4_/graphene oxide. The loss of activity in reuse can be associated with the agglomeration and/or natural deactivation of the enzyme rather than enzyme leakage, protein denaturation and inhibition [33].

## 3. Conclusions

Commercial cellulase Cellic CTec2 which was immobilized by entrapment in sol–gels showed efficient biocatalytic properties for the conversion of cellulose to glucose. The entrapment of the enzyme blend into a tailored sol–gel matrix resulted in stable biocatalysts with good catalytic efficiency. Furthermore, deposition of the sol–gel-entrapped biocatalyst onto the magnetic nanoparticles improved the possibility of the enzyme’s reuse. The tailoring of the immobilization parameters such as the nature and ratio of the silane precursors, the nature of the immobilization additives and the protein loading, allowed for the optimization of the catalytic properties of the sol–gel and magnetic sol–gel biocatalysts. The best immobilized biocatalysts were obtained with the use of a ternary silane precursor system VTMOS:PhTMOS:TMOS at a 0.4:1.6:1 molar ratio and also with the deposition of it on the magnetic particles. The sol–gel-immobilized cellulase was thoroughly characterized and displayed a good temperature and pH stability. Multiple uses of magnetic sol–gel-immobilized cellulase on a microcrystalline cellulose substrate demonstrated the capability to retain more than 40% of its residual activity after seen reuse cycles, thus opening up the possibility to investigate the catalytic performance of this new series of immobilized cellulase biocatalysts on real biomass.

## 4. Materials and Methods

### 4.1. Materials

Celulase enzyme blend Cellic CTec2 was purchased from Sigma-Aldrich. Microcrystalline cellulose Avicel PH101 (Sigma-Aldrich, Burlington, MA, USA), carboxymethylcellulose and phenol were products of Fluka. Glucose (Merck, Rahway, NJ, USA), 3,5-dinitrosalicylic acid (Merck, Rahway, NJ, USA), Coomassie Brilliant Blue G-250 (Merck, Rahway, NJ, USA), 98% phosphoric acid (Merck, Rahway, NJ, USA), bovine serum albumin (Merck, Rahway, NJ, USA) and fluorescein isothiocyanate (Merck, Rahway, NJ, USA) were of analytical grade and have been used in the state that they were in when they were purchased.

Silane precursors tetramethoxysilane (TMOS, 98%), methyltrimethoxysilane (MeTMOS, 98%) and vinyltrimethoxysilan (VTMOS, 99%) were bought from Merck; ethyltrimethoxysilane (EtTMOS, 98%), propyltrimethoxysilane (PrTMOS, 98%), *iso*-butyl-trimethoxysilane (*i*-BuTMOS, 98%) and phenyltrimethoxysilane (PhTMOS, 99%) were from Sigma-Aldrich, while octyltrimethoxysilane (OcTMOS, 98%) was from Fluka. Polyethylene glycol 20,000 (PEG; Fluka), Tween 80 surfactant (Spectrum 3D), absolute ethanol (Merck), sodium fluoride (Sigma-Aldrich), 2-propanol (Merck) and hexane (Merck) were used as reagents and additives for sol–gel immobilization. Glacial acetic acid was purchased locally.

The ionic liquids 1-octyl-3-methyl-imidazolium tetrafluoroborate (OmimBF_4_), 1-butyl-3-methyl-imidazolium hexafluorophosphate (BmimPF_6_) and 1-ethyl-3-methylimidazolium acetate (EmimAc) were products of Merck.

### 4.2. Immobilization Procedures

#### 4.2.1. Sol–Gel Entrapment Procedure and Enzyme Immobilization Yield

The immobilization procedure for the sol–gel entrapment of enzymes, which was used in this study, is a modified version of the one that was previously reported by our group [23]. Briefly, a certain volume of cellulase, depending on the enzyme loading that was used in this study, was added to 0.05 M sodium acetate buffer pH 4.8, and it was mixed with 200 μL additive (PEG20000, ionic liquid or TWEEN 80), 100 μL 1 M NaF solution and 200 μL isopropyl alcohol. The mixture was kept on the magnetic stirrer for 30 min (or 1 min) for homogenization, then the silane precursors were introduced while they were agitating until gelation occurred. The gel that was obtained was kept for 24 h at room temperature for complete polymerization to occur. The wet gel was washed with 2-propanol, distilled water, 2-propanol and n-hexane, and it was vacuum-filtered through a glass Buchner funnel (G3 porosity). The product that was obtained was kept for 24 h at room temperature and then, in a vacuum oven at a temperature of 25 °C for another 24 h (100 mbar vacuum level). The final xerogel was crushed in a mortar and stored under a refrigeration condition (4 °C). The washing filtrate was tested for proteins using the Bradford protein assay [51].

The efficiency of the immobilization process was evaluated in terms of protein *immobilization yield (%)*, which was calculated as a percentage of immobilized protein and protein subjected to immobilization.

*Entrapped protein* loading was expressed as mg of immobilized protein per ·g biocatalyst (mg·g biocatalyst^−1^).

A leaching test was also carried out to determine the possible loss of enzymes by diffusion in the solution, as described by [52]. Enzymatic activity was not detected in the samples by this assay.

#### 4.2.2. Magnetic Nanoparticles Synthesis Procedure

The magnetic nanoparticles MPs (code names MP1 and MP2) that were used in this study were synthesized in our laboratory by chemical coprecipitation of Fe^3+^ and Fe^2+^ salts in the presence of an excess of ammonia, according to [53]. Briefly, FeSO_4_ was dissolved in distilled water and mixed with a FeCl_3_ solution of appropriate concentration to yield a Fe^3+^/Fe^2+^ ratio of 1.7 since the synthesis was carried out in the presence of oxygen. This mixture was then heated to 80 °C under vigorous mechanical stirring and precipitated with ammonia solution, and the stirring was continued for another 15 min. The precipitate that was obtained was washed several times with distilled water to remove residual salts and dried under vacuum.

The magnetic diameters of the particles were 5 nm and 7 nm for MP1 and MP2, respectively, while their saturation magnetization was 70 emu g^−1^ and 60 emu g^−1^, respectively; this method is described in Section 4.6.5. The hydrodynamic diameters of the particles that were determined by DLS analysis (described in Section 4.6.6.) of the aqueous suspension of the magnetic nanoparticles were 408 nm and 187 nm for MP1 and MP2, respectively (Figure S3 in the Supplementary Materials).

#### 4.2.3. Sol–Gel Magnetic Immobilization Procedure

The sol–gel magnetic immobilization procedure was identical to the sol–gel entrapment procedure that is described in Section 4.2.1, until the onset of gelation. Next, 0.5 g of magnetic nanoparticles were added to the gelling mixture. Subsequently, the obtained magnetic gel (MSG) was processed as described above.

### 4.3. Catalytic Efficiency of the Native and Immobilized Cellulase

Cellulase activity expressed which was as catalytic efficiency was determined according to the original reducing sugar analysis method [54] that was used and reported in our previous work [17]. Briefly, 5 µL of cellulase (0.350 mg protein) or 50 mg immobilized biocatalyst of was incubated with 2% (*w*/*v*) carboxymethyl-cellulose (CMC) in 1 mL 0.05 M sodium acetate buffer, with a pH of 4.8, in a Thermomixer (Eppendorf AG, Hamburg, Germany) at 50 °C for 30 min. The reduced sugars that were released were measured spectrophotometrically at 575 nm (Agilent UV-VIS Cary 60 spectrophotometer) with the 3,5-dinitrosalicylic acid (DNS) method [55], and were expressed as glucose equivalent using a standard calibration curve.

The *catalytic efficiency* of the biocatalysts was assessed as µmol·min^−1^·mL biocatalyst^−1^ or (U·mL^−1^) for native cellulase and µmol·min^−1^·g biocatalyst^−1^ or (U·g^−1^) for the immobilized biocatalyst, with 1 U of enzymatic activity being defined as the amount of enzyme that catalyzed the release of 1.0 µmol reducing sugars per time unit (1 min) under the assay conditions. In this work, the term ‘‘efficiency’’ was used instead of ‘‘activity’’ because enzymatic activity is related to the initial reaction rate of the enzyme-catalyzed reaction. 

The *specific activity* of the biocatalysts was expressed as U total sugar that was released per g of protein (µmol·min^−1^·g protein^−1^).

Studies on the influence of temperature and pH on the catalytic performance of the immobilized biocatalysts studies have been carried out under the same experimental conditions, except for the studied parameter which was set at the proper value. The thermal profile was assessed by thermostating 2% (*w*/*v*) CMC substrate in 0.05 M sodium acetate buffer, with a pH of 4.8, at the appropriate temperature in the 50–90 °C range, which was followed by the addition of 5 µL native cellulase or 50 mg immobilized cellulase and incubation for 30 min at the same temperature.

The pH study was carried out in solutions of 0.05 M acetate buffer, at 50 °C and pH values of 4.2, 4.8, 5.2, 6.0 and 6.8, respectively. 

### 4.4. Enzymatic Hydrolysis of Cellulose

The hydrolysis of 10 mg·mL^−1^ microcrystalline cellulose (Avicel PH101) in 0.05 M sodium acetate buffer (pH 4.8) catalyzed by 6 µL·mL^−1^ native cellulase Cellic CTec2 (containing 0.417 mg protein·mL^−1^) or 50 mg immobilized biocatalyst was performed in 15 mL glass vials with lids using a Thermomixer (Eppendorf AG, Hamburg, Germany) at 750 strokes/min and 50 °C for 24 h. Glass vials with the reaction mixture were centrifuged at 6000× *g*, 10 min at 25 °C, then, the supernatant was analyzed for total released sugars using the 3,5-dinitrosalicylic acid (DNS) method [55], and this was expressed as glucose equivalent using a standard calibration curve.

The *hydrolysis yield (%)* was calculated as the ratio between the total amount of sugars expressed as glucose released after enzymatic hydrolysis (g) and the 1.11 g of glucose released by the complete hydrolysis of 1 g of cellulose.

The *sugar productivity* expressed as mg glucose per mL of reaction medium was obtained by converting 1 g of substrate after 24 h of reaction at 50 °C in the enzymatic hydrolysis of microcrystaline cellulose Avicel using native or immobilized cellulase biocatalysts.

All of the experiments were performed in duplicate, and all of the samples were measured in triplicate. Results that are given in tables and figures are average values, as the standard deviation for repeated measurements did not exceed 3%.

### 4.5. Reuse of the Magnetic Sol–Gel Biocatalyst

The reusability of the magnetic sol–gel biocatalyst was tested on both the cellulosic substrate CMC and Avicel PH101. For this purpose, 50 mg was subjected to catalytic activity assays according to the method described above. After the activity assay, the biocatalyst was collected with an external magnet, washed with a buffer solution, and the same experiment was repeated with a fresh CMC substrate solution.

As for the hydrolysis of microcrystaline cellulose (Avicel PH101), 50 mg magnetic sol–gel immobilized biocatalyst was added to 5 mL of 0.05 M sodium acetate buffer (pH 4.8), containing 10 mg·mL^−1^ microcrystaline cellulose (Avicel PH101), and it was incubated at 50 °C. After 24 h of enzymatic hydrolysis, the magnetic immobilized enzyme was separated using an external magnet, washed with buffer solution, and the same experiment was repeated with fresh Avicel substrate. The total reducing sugars were assayed by the DNS method, as previously described, and were expressed as glucose equivalent.

All of the experiments were performed in duplicate, and all of the samples were measured in triplicate. Results that are given in tables and figures are average values, as the standard deviation for repeated measurements did not exceed 3%.

### 4.6. Characterization of the Biocatalysts 

#### 4.6.1. Scanning Electron Microscopy (SEM)

The morphology of the investigated materials was investigated by scanning electron microscopy (SEM: Quanta FEG 250, FEI, Hillsboro, OR, USA) along with energy dispersive X-ray spectroscopy analysis (EDX with Apollo SSD: detector, EDAX Inc., Mahwah, NJ, USA). The SEM was operated in low-vacuum mode, using 5 kV and a 1.5 spot size to avoid sample charging. EDX was used for chemical characterization and quantification by positioning the samples at a working distance of about 10 mm. The measurements were performed on 8 μm^2^ areas and to increase the accuracy of the measurements of 5 particles from each material that was investigated.

#### 4.6.2. Fluorescence Microscopy (FM)

To investigate the distribution of the enzyme in the sol–gel matrix, the fluorescence microscopy technique was performed using an inverted microscope; Leica DMI4000B (Leica, Munich, Germany) was used. For this purpose, a coupling reaction of Cellic CTec 2 cellulase with a compound showing fluorescence such as fluorescein isothiocyanate (FITC) was performed according to the PierceTM FITC kit. The removal of unbound FITC from the obtained solution was carried out by centrifugation in a centrifuge tube with an Amicon Ultra-4 filter (cut off 10 KDa) and repeated washings with distilled water until the collected fractions had absorbance at a wavelength of 493 nm of about 0.1. The obtained solution (containing FITC-labeled enzyme or enzyme-FITC complex) was concentrated to 10 mg protein·mL^−1^ by centrifugation in an Amicon Ultra-4 filter tube and used for immobilization by sol–gel entrapment. For comparison, a blank sol–gel matrix without the enzyme-FITC complex was also prepared.

#### 4.6.3. Fourier Transform Infrared Spectroscopy (FTIR)

Fourier transform infrared (FTIR) spectroscopy analyses of the samples were performed in attenuated total reflectance (ATR) mode using a Bruker Vertex 70 (Bruker Daltonik GmbH, Germany) spectrometer that was equipped with a Platinum ATR, Bruker Diamond Type A225/Q. Sample spectra were collected in the range of 4.000−400 cm^−1^ at 64 scans/min with a resolution of 4 cm^−1^.

#### 4.6.4. Thermal Analysis (TGA/DTA)

Thermogravimetric and differential thermal analysis (TGA/DTA) were recorded using a TG 209 F1 Libra thermogravimetric analyzer (Netzsch, Selb, Germany) operating at a resolution of 0.1 µg under nitrogen atmosphere conditions. Thermogravimetric curves were recorded from 30 to 1000 °C, with a heating rate of 10 °C·min^−1^. The average sample mass was 5.0 ± 0.2 mg. The samples were tested in open alumina crucibles (average mass 190 ± 1.0 mg).

#### 4.6.5. Vibrating Sample Magnetometry (VSM). Magnetogranulometry

The full magnetization curves, including the initial susceptibility and saturation magnetization of the magnetic nanoparticles, were determined using a VSM 880 vibrating sample magnetometer (ADE Technologies, Pensacola, FL, USA) at room temperature in the field range of 0–1000 kA m^−1^. The magnetization data were used for magnetogranulometry analysis which consisted of determination of the magnetic diameter distribution from nonlinear regression of the experimental data according to [53,56].

#### 4.6.6. Dynamic Light Scattering (DLS)

The mean hydrodynamic diameter of the iron oxide particles was determined at 25 ± 0.1 °C by dynamic light scattering (DLS) using the NanoZS device from Malvern (UK) which was operating in backscattering mode at an angle of 173°. The concentration of the dispersions was set to give an optimal intensity of ~100 counts per second. The diluted samples were homogenized in an ultrasonic bath for 10 s prior to the measurements, after which, 50 s of relaxation was allowed. The size was measured in a disposable zeta cell (DTS 1070). Cumulative analysis was used to calculate the average hydrodynamic sizes. In the case of unstable, coagulating systems, the measurable hydrodynamic size increased over time.

## Figures and Tables

**Table 1:** Influence of the binary silica network on the immobilization yield and catalytic efficiency of the immobilized biocatalysts by their entrapment in sol–gel and magnetic sol–gel.

Biocatalyst	Silane Type (Molar Ratio)	MP Type	Immobilization Yield,	Entrapped Protein Loading,	Catalytic Efficiency *,	Specific Activity,
			%	mg·g Biocatalyst^−1^	µmol·min^−1^·g Biocatalyst^−1^	µmol·min^−1^·g Protein^−1^
SG1	MeTMOS:TMOS (1:1)		>99	20.6	5.9	288
SG2	EtTMOS:TMOS (1:1)		99	19.3	2.3	119
SG3	VTMOS:TMOS (1:1)		99	20.0	4.8	238
SG4	PrTMOS:TMOS (1:1)		98	18.8	5.6	298
SG5	*i*-BuTMOS:TMOS (1:1)		77	18.2	6.7	366
SG6	PhTMOS:TMOS (1:1)		84	14.2	2.2	153
M1-SG6	PhTMOS:TMOS (1:1)	MP1	55	5.3	1.5	277
M2-SG6	PhTMOS:TMOS (1:1)	MP2	63	5.7	0.6	101
SG7	OcTMOS:TMOS (1:1)		95	8.5	3.7	216

* CMC assay; MP—magnetic particles.

**Table 2:** Influence of the ternary silica network on the immobilization yield and catalytic efficiency of immobilized biocatalysts by their entrapment in sol–gel and magnetic sol–gel.

Biocatalyst	Silane Type (Molar Ratio)	MP Type	Immobilization Yield,	Entrapped Protein Loading,	Catalytic Efficiency *,	Specific Activity,
			%	mg·g Biocatalyst^−1^	µmol·min^−1^· g Biocatalyst^−1^	µmol·min^−1^· g Protein^−1^
SG8	MeTMOS:PhTMOS:TMOS (0.4:1.6:1)		44	7.6	6.6	873
SG9	VTMOS:PhTMOS:TMOS (0.4:1.6:1)		52	8.9	6.7	757
M1-SG9	VTMOS:PhTMOS:TMOS (0.4:1.6:1)	MP1	26	2.5	5.5	2167
M2-SG9	VTMOS:PhTMOS:TMOS (0.4:1.6:1)	MP2	21	2.0	5.4	2635
SG10	VTMOS:PhTMOS:TMOS (1:1:1)		68	12.3	5.5	446
SG11	VTMOS:PhTMOS:TMOS (1.6:0.4:1)		90	18.3	5.9	322

* CMC assay; MP—magnetic particles.

**Table 3:** EDS quantification of the immobilized biocatalysts in weight percentages (wt. %).

Biocatalyst	Silane Type (Molar Ratio)	C	N	O	F	Na	Si	C/Si
SG6	PhTMOS:TMOS (1:1)	49.37	1.73	39.71	0.86	0.28	8.05	6.13
SG9	VTMOS:PhTMOS:TMOS (0.4:1.6:1)	47.38	1.67	36.31	0.32	0.23	14.08	3.37
SG10	VTMOS:PhTMOS:TMOS (1:1:1)	37.54	2.17	44.07	0.21	0.25	15.77	2.38
SG11	VTMOS:PhTMOS:TMOS (1.6:0.4:1)	39.98	1.76	40.38	0.47	0.25	17.16	2.33

**Table 4:** Thermal behavior of biocatalysts that were obtained by the sol–gel entrapment of Cellic CTec2 cellulase.

Biocatalyst	Weight Loss, %			Residual Mass, %
	30–280 °C	280–530 °C	530–990 °C	
Blank-SG6 matrix	2.80	5.32	20.18	71.40
SG6	3.82	5.43	16.46	71.73
Blank-SG9 matrix	2.62	6.33	11.30	79.42
SG9	3.05	6.46	18.12	70.20
SG10	2.16	4.34	6.76	84.59
SG11	2.28	3.79	5.14	86.68

**Table 5:** Influence of protein loading on the enzymatic hydrolysis of microcrystalline cellulose Avicel-PH101, at 24 h reaction time and 50 °C, as catalyzed by immobilized biocatalysts which were obtained by their entrapment in sol–gel and magnetic sol–gel.

Biocatalyst	Silane Type (Molar Ratio)	MP Type	Initial Protein Dosing,	Entrapped Protein Loading,	Sugar Productivity,
			mg·mmol Silane^−1^	mg·g Biocatalyst^−1^	mg·mL^−1^·g Substrate^−1^
SG6	PhTMOS:TMOS (1:1)		1.66	14.2	74
			3.33	29.3	104
			5.00	43.0	111
SG9	VTMOS:PhTMOS:TMOS (0.4:1.6:1)		1.66	8.9	58
			3.33	28.5	108
			5.00	NG *	-
M1-SG9	VTMOS:PhTMOS:TMOS (0.4:1.6:1)	MP1	1.66	2.5	22
			3.33	11.2	149
M2-SG9	VTMOS:PhTMOS:TMOS (0.4:1.6:1)	MP2	1.66	2.0	20
			3.33	11.8	118

* NG—no gelling; MP—magnetic particles.

## Data Availability

The data presented in this study are available on request from the corresponding authors.

## Supplementary Materials

The following supporting information can be downloaded at: https://www.mdpi.com/article/10.3390/gels8100626/s1, Figure S1: Magnetization curves of the magnetic nanoparticles MP1 and MP2.; Figure S2: FT-IR spectra of: (**a**) magnetic support MP2 and (**b**) cellulase biocatalyst (M2-SG9), obtained by magnetic sol–gel immobilization method with silane precursors VTMOS: PhTMOS:TMOS (0.4:1.6:1) and MP2. Inset: enlarged picture of the 400–1200 cm^−1^ region, showing the presence of the absorption bands specific for the maghemite; Figure S3. Hydrodynamic size distributions of the magnetic nanoparticles MP1 and MP2 as determined by DLS.
