# Supplementary material for: Enhancement of the Catalytic Performance and Operational Stability of Sol-Gel-Entrapped Cellulase by Tailoring the Matrix Structure and Properties
Source: Gels. 2022 Oct 1;8(10):626. doi: 10.3390/gels8100626 (PMC9602319; doi:10.3390/gels8100626)
Supplement: Supplementary file 1 [file gels-08-00626-s001.zip › gels-1930411-supplementary.pdf]

Enhancement of the Catalytic Performance and Operational Stability of Sol-Gel Entrapped Cellulase by Tailoring the Matrix Structure and Properties

Corina Vasilescu [1,2], Simona Marc [1,3], Iosif Hulka [4] and Cristina Paul [1,*]

1 Biocatalysis Group, Department of Applied Chemistry and Engineering of Organic and Natural Compounds, Faculty of Industrial Chemistry and Environmental Engineering, Politehnica University Timisoara, Carol Telbisz 6, 300001 Timisoara, Romania;

2 Laboratory of Magnetic Fluids, Center for Fundamental and Advanced Technical Research, Romanian Academy Timisoara Branch, Mihai Viteazu 24, 300223 Timisoara, Romania;

3 Faculty of Veterinary Medicine, University of Life Sciences ''King Mihai I'' from Timisoara, Calea Aradului 119, 300645 Timisoara, Romania;

4 Research Institute for Renewable Energy, Politehnica University Timisoara, Gavril Musicescu 138, 300501 Timisoara, Romania

* Correspondence: cristina.paul@upt.ro

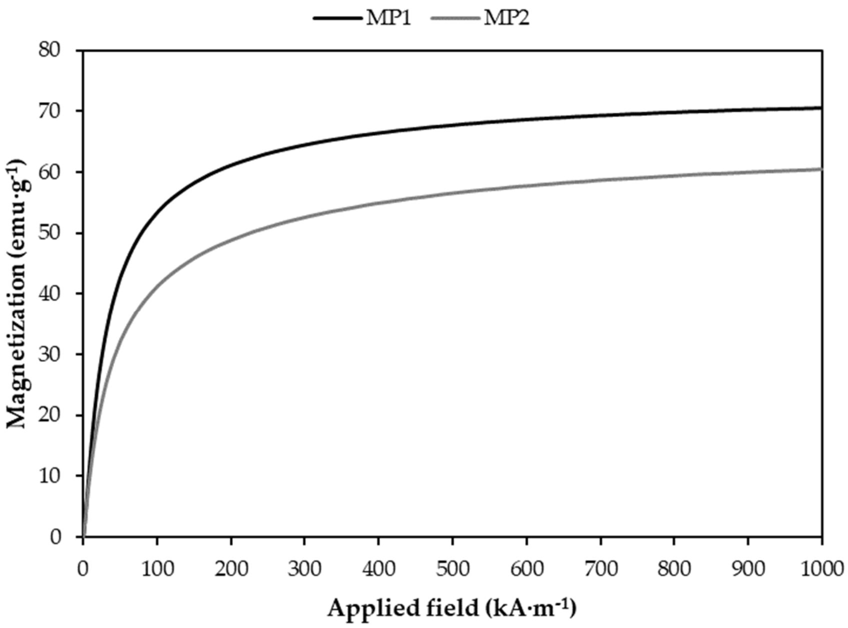

Figure S1. Magnetization curves of the magnetic nanoparticles MP1 and MP2.

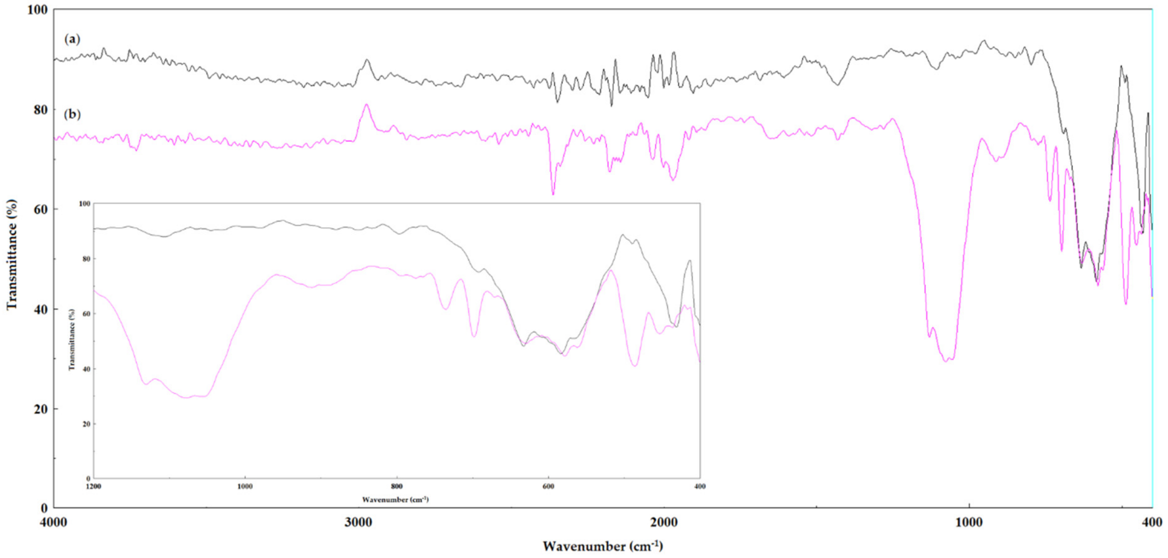

Figure S2. FT-IR spectra of: (**a**) magnetic support MP2 and (**b**) cellulase biocatalyst (M2-SG9), obtained by magnetic sol-gel immobilization method with silane precursors VTMOS: PhTMOS:TMOS (0.4:1.6:1) and MP2. Inset: enlarged picture of the 400-1200 cm^{-1} region, showing the presence of the absorption bands specific for the maghemite.

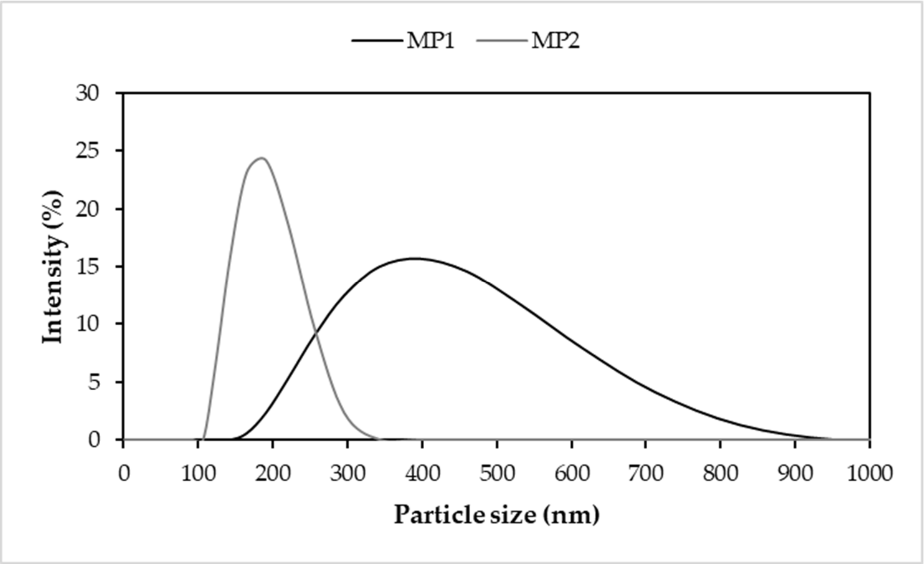

Figure S3. Hydrodynamic size distributions of the magnetic nanoparticles MP1 and MP2 as determined by DLS.
